# Intracranial Efficacy of Systemic Therapy in Patients with Asymptomatic Brain Metastases from Lung Cancer

**DOI:** 10.3390/jcm12134307

**Published:** 2023-06-27

**Authors:** Min-Gwan Sun, Sue Jee Park, Yeong Jin Kim, Kyung-Sub Moon, In-Young Kim, Shin Jung, Hyung-Joo Oh, In-Jae Oh, Tae-Young Jung

**Affiliations:** 1 Department of Neurosurgery, Chonnam National University Medical School, Chonnam National University Hwasun Hospital, Hwasun 58128, Republic of Koreasuejee1991@naver.com (S.J.P.); moonks@chonnam.ac.kr (K.-S.M.);; 2 Department of Internal Medicine, Chonnam National University Medical School, Chonnam National University Hwasun Hospital, Hwasun 58128, Republic of Korea

**Keywords:** brain metastases, chemotherapy, local treatment, lung cancer, progression-free survival, targeted therapy

## Abstract

There has been controversy over whether to radiologically follow up or use local treatment for asymptomatic small-sized brain metastases from primary lung cancer. For brain tumors without local treatment, we evaluated potential factors related to the brain progression and whether systemic therapy controlled the tumor. We analyzed 96 patients with asymptomatic small-sized metastatic brain tumors from lung cancer. These underwent a radiologic follow-up every 2 or 3 months without local treatment of brain metastases. The pathologies of the tumors were adenocarcinoma (*n* = 74), squamous cell carcinoma (*n* = 11), and small cell carcinoma (*n* = 11). The primary lung cancer was treated with cytotoxic chemotherapy (*n* = 57) and targeted therapy (*n* = 39). Patients who received targeted therapy were divided into first generation (*n* = 23) and second or third generation (*n* = 16). The progression-free survival (PFS) of brain metastases and the overall survival (OS) of patients were analyzed depending on the age, tumor pathology, number, and location of brain metastases, the extent of other organ metastases, and chemotherapy regimens. The median PFS of brain metastases was 7.4 months (range, 1.1–48.3). Targeted therapy showed statistically significant PFS improvement compared to cytotoxic chemotherapy (*p* = 0.020). Especially, on univariate and multivariate analyses, the PFS in the second or third generation targeted therapy was more significantly improved compared to cytotoxic chemotherapy (hazard ratio 0.229; 95% confidence interval, 0.082–0.640; *p* = 0.005). The median OS of patients was 13.7 months (range, 2.0–65.0). Univariate and multivariate analyses revealed that the OS of patients was related to other organ metastases except for the brain (*p* = 0.010 and 0.020, respectively). Three out of 52 patients with brain recurrence showed leptomeningeal dissemination, while the recurrence patterns of brain metastases were mostly local and/or distant metastases (94.2%). Of the 52 patients who relapsed, 25 patients received local brain treatment. There was brain-related mortality in two patients (2.0%). The intracranial anti-tumor effect was superior to cytotoxic chemotherapy in the treatment of asymptomatic small-sized brain metastases with targeted therapy. Consequently, it becomes possible to determine the optimal timing for local brain treatment while conducting radiological follow-up for these tumors, which do not appear to increase brain-related mortality. Furthermore, this approach has the potential to reduce the number of cases requiring brain local treatment.

## 1. Introduction

Brain metastases represent the most common intracranial neoplasms in adults and are the primary cause of neurologic complications resulting from systemic cancers. Brain metastases are found in approximately 10–20% of patients with non-small cell lung cancer (NSCLC) at the time of the first diagnosis. Moreover, approximately 40% of all patients develop brain metastases during the course of their disease [1]. Radiation therapy (RT), either via stereotactic radiosurgery (SRS) or whole brain (WBRT), remains the primary modality of treatment for metastatic brain tumors [2].

Left untreated, afflicted patients exhibit only a 3-month median survival. However, there has been a notable increase in systemic therapy options for patients with intra-parenchymal metastases over the last decade, which has dramatically improved both the progression-free survival (PFS) and overall survival (OS) of patients with these cancers [3]. The discovery of actionable driver mutations has led to the development of tyrosine kinase inhibitors (TKIs) that target the epidermal growth factor receptor (EGFR) mutations and anaplastic-lymphoma-kinase (ALK) rearrangements. Currently, second- and third-generation EGRK and ALK targeted therapy with higher molecular specificity and the ability to cross the central nervous system (CNS) barriers are being developed. Despite this improvement in longevity, the quality of life for the patient with brain metastasis remains poor and secondary to neurologic and cognitive impairment [4]. The management of asymptomatic brain metastases has been a subject of ongoing research, with systemic therapy playing a crucial role in treatment strategies. Understanding the intracranial efficacy of systemic therapy in these patients is essential for optimizing treatment outcomes and improving patient quality of life.

However, invasive treatment may not be necessary if a way to stop the progression in patients with asymptomatic brain metastases exists. Indeed, several targeted therapies were invented for brain metastases resulting from lung cancer which can pass through the blood brain barrier (BBB). Yet, there were cases where only targeted therapy was maintained in asymptomatic tiny brain metastases. Therefore, this article aims to explore the intracranial efficacy of systemic therapy in patients with asymptomatic brain metastases from lung cancer without brain local treatment, shedding light on the effectiveness of different treatment approaches and providing insights for clinical decision-making. By examining the existing literature and available evidence, this study aims to contribute to the growing body of knowledge in this field and potentially guide future treatment guidelines and practices. Moreover, this article aims to analyze the factors that influence PFS and OS in cases of small-sized asymptomatic brain metastases from lung cancer.

## 2. Materials and Methods

### 2.1. Data Collection

Retrospectively, single-center (Chonnam National University Hwasun Hospital) data were collected from November 2005 to September 2018; a total of 1516 patients with brain metastases resulting from lung cancer were treated. In our center, when administering chemotherapy to patients with asymptomatic brain metastases, we initially conduct radiological follow-ups without implementing any local treatments such as surgery, radiotherapy, or gamma knife surgery (GKS). This approach allows us to observe the response to chemotherapy. This retrospective study was approved by our Institutional Review Board, and the need for written informed consent was waived due to the retrospective design of this study. The inclusion criteria were as follows: patients who were treated with systemic therapy for lung cancer, radiologic follow-up by brain MRI with gadolinium enhancement every 2 or 3 months without local treatment of brain metastases, asymptomatic patients with tumors <1 cm and with or without mild edema, a Karnofsky Performance score (KPS) score of ≥70, and no previous history of treatment for brain metastases. Using these inclusion criteria, a total of 96 patients were included in this study.

Data were collected by a neurosurgeon and MRIs were interpreted by a neuro-radiologist. The KPS scale (points 0–100) was used to assess disease progression and how the disease and treatments affected the activities of daily living. This was then used to determine the appropriate treatment and for estimating the prognosis. Due to the durability of chemotherapy, patients with a KPS score below 70 were excluded.

We all included synchronous and metachronous brain metastases with or without chemotherapy. The characteristics of the metastatic tumors were investigated. All tiny tumors were defined as 1 mm to be easily understood. Treatment methods and prognoses were also investigated. Systemic therapy on lung cancer was performed using the molecular profiles of all individual patients. Regarding the driver mutations of EGFR and ALK, the targeted therapy was administration. In cases where there were no driver mutations, traditional cytotoxic chemotherapy was used. Here we divided treatments into standard cytotoxic chemotherapy (*n* = 57, 59.3%) and molecular targeted therapy (*n* = 39, 40.6%). The EGFR mutation target agents were classified whereby the first-generation drug was gefitinib (*n* = 10) or erlotinib (*n* = 12), the second-generation drug was afatinib (*n* = 11), and the third-generation drug was osimertinib (*n* = 4). Similarly, according to ALK rearrangement, the first-generation drug was crizotinib (*n* = 1), the second-generation drugs were ceritinib (*n* = 0), alecitinib (*n* = 0), and brigatinib (*n* = 0), and the third-generation drug was lorlatinib (*n* = 1). The 39 patients who were treated with targeted therapy were divided into 1st-generation (*n* = 23, 23.9%) and 2nd- and 3rd-generation (*n* = 16, 16.6%) according to the above criteria. Finally, the treatment duration was measured for each.

In this study, the evaluation of intracranial tumor progression was evaluated by RECIST (Response Evaluation Criteria in Solid Tumors). Local recurrence was defined as a recurrence at the same site with an increased diameter of more than 120% compared to the previous tumor size. Distance recurrence was defined as a metastasis spatially separated from the previously detected lesion. Dissemination was defined as recurrence in the ventricles, subarachnoid space, and spinal cord diagnosed by imaging studies or tumor cells on cerebrospinal fluid cytology.

### 2.2. Statistical Analysis

The effects of single variables on PFS and OS were determined by univariate and multivariate analyses. The single variables were age, tumor pathology, recurrence pattern, location and number of metastases, treatment duration, and anti-cancer regimen.

The prognosis was evaluated with PFS and OS, with the median value and the range from the minimum to maximum. PFS was focused on intracranial lesions, not on extracranial lesions. PFS was measured from the date of brain metastases diagnosis to the date of either the recurrence or last follow-up visit. OS was measured for the period from the date of diagnosis to the date of either death or their last follow-up.

The survival probability was calculated using the Kaplan–Meier method, and comparisons were made using a log-rank test. The variables were examined using a Cox proportional hazard analysis model to identify independent predictors of survival. On multivariate analysis, we conducted an analysis on factors that had meaningful *p*-values or were aligned with the purpose of the paper. A linear regression analysis was performed to assess the relationship between the progression-free survival (PFS) of brain metastases and the duration of targeted therapy. All statistical analyses were carried out with a significance level of *p* < 0.05 using SPSS 21.0 (SPSS, Chicago, IL, USA).

## 3. Results

### 3.1. Characteristics

The characteristics of the 96 patients are summarized in Table 1. The average age was 66 years (range, 35–85 years). The pathological types of lung cancer were adenocarcinoma (*n* = 74, 77%), squamous cell carcinoma (*n* = 11, 11.5%), and small cell lung carcinoma (*n* = 11, 11.5%). The presence or absence of other organ metastases (except brain) was investigated at the time of diagnosis. Here, six patients were identified without any other metastases and 90 patients possessed other organ metastases with brain metastases. The location alongside the number of brain metastases were divided into supratentorial (*n* = 66, 68.7%) or infratentorial (*n* = 8, 8.3%) or both (*n* = 22, 23%) and numbered as 1 (*n* = 47, 48.9%), 2 to 5 (*n* = 24, 25%), and over 5 (*n* = 25, 26%). The median number was 2 (range, 1–93), while the maximum tumor size was measured in individual patients, and the average size was 3.7 mm (range, 1–9.5).

### 3.2. PFS-Related Factors

**Figure 1:**
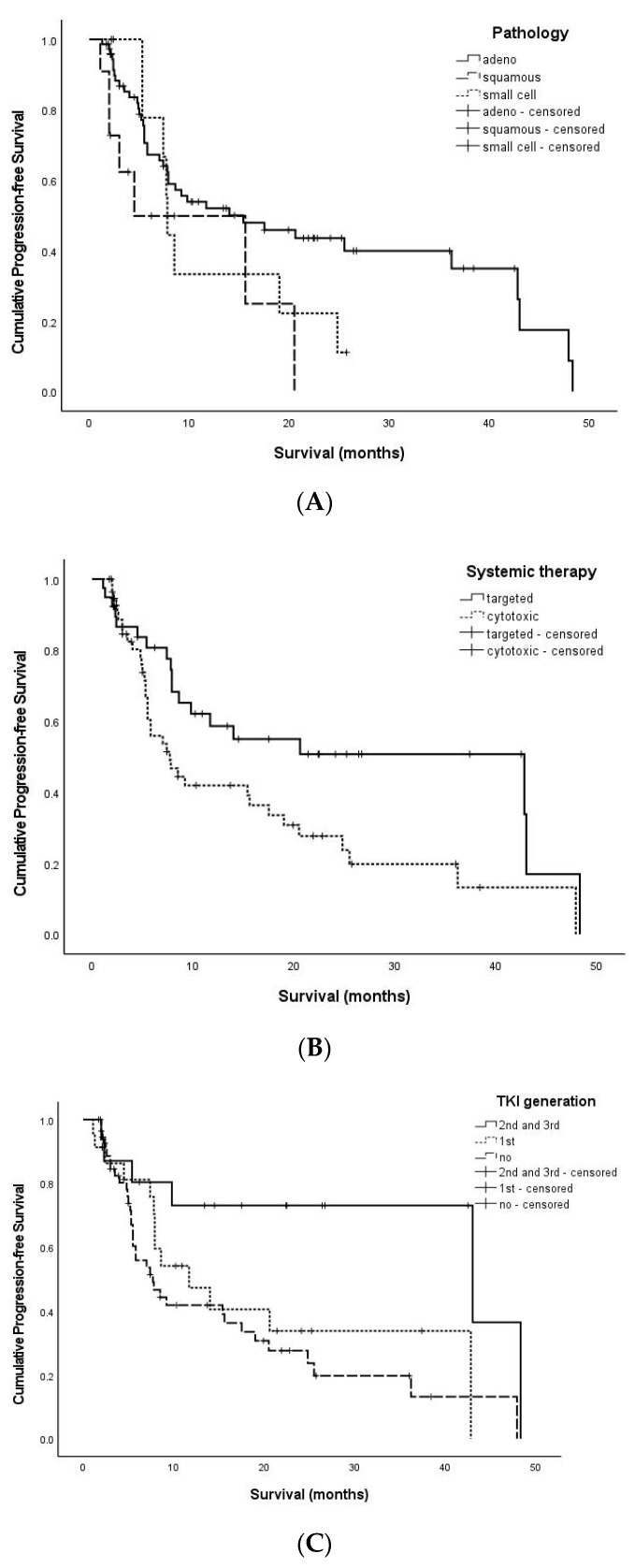
The Kaplan–Meier curve of PFS-related factors. (**A**): Adenocarcinoma revealed a higher PFS than squamous cell carcinoma or small cell carcinoma (*p* = 0.069). (**B**): Targeted therapy showed an improved PFS compared to cytotoxic chemotherapy (*p* = 0.048). (**C**): The second- and third-generation targeted therapy exhibited an improved PFS compared to either the first-generation targeted therapy or cytotoxic chemotherapy (*p* = 0.016) (adeno: adenocarcinoma, squamous: squamous cell carcinoma, small cell: small cell carcinoma, targeted: targeted therapy, cytotoxic: cytotoxic chemotherapy, TKI: tyroisine kinase inhibitor).

After diagnosis with asymptomatic brain metastases, the median PFS of the 96 patients was 7.4 months (range, 1.1–48.3). The univariate analysis findings are summarized in Table 2. There was no statistical significance in the age distribution (*p* = 0.861). Depending on the pathologies, the median PFS was 7.9 months (1.3–48.3) for adenocarcinoma, 3.9 months (1.1–20.5) for squamous cell carcinoma, and 7.7 months (2.2–25.7) for small cell carcinoma (*p* = 0.069) (Figure 1A). The median PFS of a single tumor was 7.4 months (1.3–48.3); the number from 2 to 5 was 6.6 months (1.1–47.9); the number over 5 was 7.9 months (1.9–36.2) (*p* = 0.857). Depending on the location, the median PFS of patients who possessed tumors at supratentorial lesions was 7.4 months (1.1–48.3); infratentorial, 8.2 months (2.0–10.9); and both was 6.8 months (1.9–47.9) (*p* = 0.614). The median value of PFS according to the presence or absence of metastases to other organs was 7.2 months (1.1–48.3) for the presence of other organ metastases, and it was 16.5 months (3.4–47.9) for the absence of other organ metastases (*p* = 0.399).

Analysis through the anti-cancer treatment method identified a median PFS of patients who were treated with cytotoxic chemotherapy of 5.5 months (1.7–47.9), while those treated with molecular targeted therapy was 10.2 months (1.1–48.3) (*p* = 0.020) (Figure 1B). Following the classification of systemic therapy as cytotoxic chemotherapy, first-generation target therapy, and second- and third-generation, the PFS of the tumors according to each treatment was analyzed. For treatment with first-generation targeted therapy, the median PFS was 7.9 months (1.1–42.8); with second- and third-generation targeted therapy, it was 16.0 months (2.0–48.3); and for cytotoxic chemotherapy, it was 5.5 months (1.7–47.9) (*p* = 0.016) (Figure 1C).

The multivariate analysis findings related to PFS are summarized in Table 3. During the multivariate analysis of PFS, we conducted an analysis of the *p*-values considering the presence of three significant factors: the presence of other organ metastases, the type of pathology, and the type of TKI generation. The second and third generations of target therapy agents showed improved PFS (hazard ratio, 0.229; 95% confidence interval [CI], 0.082–0.640; *p* = 0.005) compared to other clinical variables including other organ metastases and pathologies.

### 3.3. OS-Related Factors

**Figure 2:**
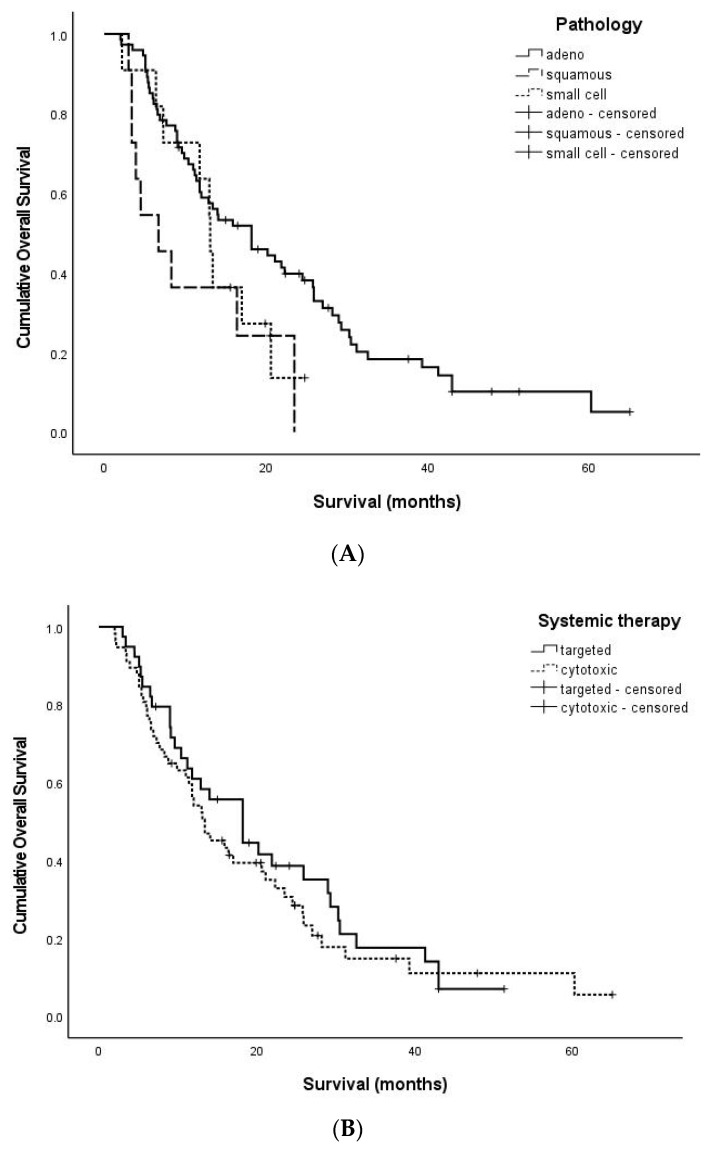
The Kaplan–Meier curve of OS-related factors. (**A**): Adenocarcinoma indicated an improved OS over either squamous cell carcinoma or small cell carcinoma (*p* = 0.032). (**B**): Targeted therapy did not show any meaningful improvement in OS compared to cytotoxic chemotherapy (*p* = 0.445) (adeno: adenocarcinoma, squamous: squamous cell carcinoma, small cell: small cell carcinoma, targeted: targeted therapy, cytotoxic: cytotoxic chemotherapy).

After diagnosis with brain metastases, the median OS was 13.7 months (range, 2.0–65.0) in 96 patients. The results of the univariate analyses related to OS are summarized in Table 2. There was not any statistically significant effect from age (*p* = 0.972), number of tumors (*p* = 0.125), tumor location (*p* = 0.240), and recurrence pattern (*p* = 0.208) on OS. Depending on the pathologies, the median OS was 15.4 months (2.0–65.0) for patients with adenocarcinoma, 6.7 months (3.0–23.5) for those with squamous cell carcinoma, and 13.1 months (2.2–24.8) for those with small cell carcinoma (*p* = 0.032) (Figure 2A). The absence of other organ metastases provides a correlation with OS (*p* = 0.010). Depending on the anti-cancer regimens, the median OS of patients who received targeted therapy was 18.2 months (3.0–51.3), while for patients who received cytotoxic chemotherapy it was 13.1 months (2.0–65.0) (*p* = 0.445) (Figure 2B). However, there was no significance from employing the targeted therapy generation (*p* = 0.401).

The results of the multivariate analyses related to OS are summarized in Table 3. During the multivariate analysis of OS, we conducted an analysis of the *p*-values considering the presence of four significant factors: the presence of other organ metastases, the type of pathology, the type of TKI generation, and the recurrence pattern. Among the clinical factors assessed, including the absence of other organ metastases, the overall survival (OS) showed improvement.

### 3.4. Management of Recurrent Brain Metastases

The recurrence patterns were classified into no recurrence (*n* = 44, 45.8%), local recurrence (*n* = 8, 8.3%), distant including local recurrence (*n* = 41, 42.7%), and dissemination (*n* = 3, 3.1%).

In the 52 cases of recurrence of brain metastases, WBRT was performed on 11 patients and GKS on 14 patients, while 27 patients were recommended for palliative treatment considering their primary cancer status.

### 3.5. A Representative Case Treated with Targeted Therapy without Local Brain Treatment

**Figure 3:**
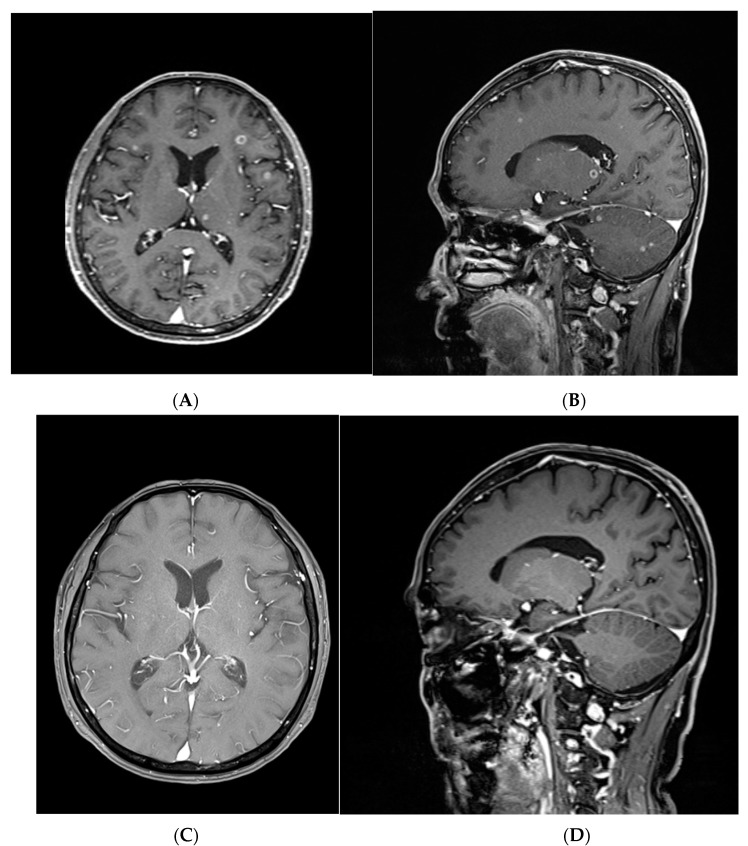
A representative case treated with targeted therapy without local brain treatment. (**A**): Multiple heterogeneously enhancing masses (maximum 0.8 cm-sized) in both the cerebral hemispheres with perilesional edema on axial T1-weighted MRI with gadolinium enhancement. (**B**): Multiple heterogeneously enhancing masses (maximum 0.2 cm-sized) in both the supratentorial and infratentorial lesions with perilesional edema on sagittal T1-weighted MRI with gadolinium enhancement. (**C**): The axial brain T1-weighted MRI with gadolinium enhancement showed complete resolution five months after afatinib treatment. (**D**): The sagittal brain T1-weighted MRI with gadolinium enhancement revealed complete resolution five months after afatinib treatment.

A 70-year-old male patient had over 40 metastatic brain tumors on the whole brain, with a maximum size of 8 mm (Figure 3A,B). This was an asymptomatic, synchronous metastatic tumor produced from an adenocarcinoma in the lung. Multiple metastatic lesions were identified in the right pleura, both lungs, the brain, both adrenal glands, and bones. Afatinib, the second-generation targeted therapy, was administered. Two months after targeted therapy without brain local treatment, a follow-up brain image highlighted a partial response to brain metastatic lesions. Five months following targeted therapy, the enhancing mass had completely regressed, and the perilesional edema had also decreased without neurological symptoms (Figure 3C,D). After 8 months, the radiologic follow-up was conducted without any recurrence. Furthermore, 9 months after targeted therapy, the patient visited the hospital due to a gait disturbance. Brain imaging was performed, which illustrated a recurrence of the brain metastases. WBRT was performed due to evidence of dissemination. After WBRT, palliative care was carried out due to the primary lung cancer status.

## 4. Discussion

Lung cancer is a highly aggressive disease associated with a poor prognosis. While systemic therapy has been employed to improve outcomes, brain metastases pose a significant challenge, contributing to reduced quality of life and dismal prognoses [5,6]. As a result, even in asymptomatic cases, routine brain magnetic resonance imaging (MRI) screenings are conducted for staging and screening purposes. Active interventions are initiated to control the disease upon detection of tiny asymptomatic brain metastases. The American Society of Clinical Oncology–Society of Neuro-Oncology–American Society for Radiation Oncology (ASCO-SNO-ASTRO) guidelines for brain metastases recommend that local therapy for asymptomatic brain metastases should not be deferred [7].

However, the treatment methods for brain metastases from lung cancer patients with low life expectancy and poor systemic conditions are limited. WBRT, SRS, and operations are viable options. Especially, WBRT has the advantage of being non-invasive and able to control the whole brain. Yet, it cannot be used multiple times and promotes delayed cognitive impairment after treatment. Thus, deferring these treatment tools as much as possible will help in improving the patient’s prognosis by avoiding the side effects of local treatments without meaningful results [7].

In order to control brain metastases, it is important to firstly control primary lung cancer. However, as is illustrated by the short survival period of lung cancer, it is difficult to control lung cancer. Pemetrexed is currently the most commonly used drug in cytotoxic chemotherapy for driver-negative adenocarcinoma of the lung. Cisplatin-pemetrexed produced a 41.9% intracranial response rate alongside a 34.9% extra-cranial response rate. Moreover, carboplatin-pemetrexed elicited a 40% intracranial response rate and a 17% extra-cranial response [8,9]. Nintedanib or ramucirumab combined with docetaxel improved the PFS and OS but did not meet the defined satisfactory expectations for the CNS response [10,11]. In cases where traditional chemotherapy yields a low intracranial response, there is a high likelihood of progression in brain metastases. If such progression occurs, it can lead to additional neurological deficits and increased costs, such as the requirement for surgery.

Recent advances in the tumor molecular analyses of lung cancer have facilitated testing for possible genetic mutations and any aberrations that drive tumor growth and proliferation. Incidences of brain metastases are higher in patients with adenocarcinomas, EGFR mutations, or ALK rearrangements [12,13]. Advancements in the molecular screening of tumors, especially EGFR and ALK, have improved patient outcomes through the development and use of targeted therapeutics. These targeted therapies were effective in improving both the PFS and OS of the patient and greatly affected the overall treatment paradigm for lung cancer. The PFS and OS were no different for small CNS diseases receiving radiation versus targeted systemic therapy alone [14,15]. Indeed, several EGFR-TKIs trials demonstrated better results than cytotoxic chemotherapy. The first-generation EGFR-TKIs trials for gefitinib or erlotinib produced a 68% response rate and a PFS of 9.6 months [16,17]. The PFS for afatinib, a second-generation EGFR-TKI, was 8.2 months, which was 5.4 months higher than cytotoxic chemotherapy groups, while the response rate was 70%. Although the use of afatinib and dacomitinib in animal experiments is known to provide a low distribution in the brain, they elicit better outcomes in patients with brain metastases compared to first-generation TKIs [18]. Osimertinib, a third-generation EGFR-TKI, provided a 91% response rate and a PSF of 15.2 months. Moreover, it provided better performance than first-generation TKIs in treating CNS metastases and in reducing the incidence of de novo CNS lesions [18,19]. New agents such as AZD3759 and epitinib (HMPL-813) that target EGFR mutations have been designed to provide excellent penetration of the CNS [5].

ALK-TKIs also show a higher intracranial control rate than cytotoxic chemotherapy. However, crizotinib, an ALK first-generation TKI, has poor penetrance to the CNS due to a pharmacokinetic resistance by the ABCB1 pumps, ultimately inducing a response rate of 56% [20,21]. Conversely, ceritinib and alectinib, ALK second-generation TKIs, show better results. Ceritinib produces an intracranial response rate of 72.7% and a PFS of 10.7 months. Alternatively, alectinib has an intracranial response rate of 54.2% [22]. Ceritinib and alectinib possess a higher inhibitory potency to the wild-type fused ALK protein, a better affinity for the secondarily mutated proteins, an improved penetrance to the CNS, and a lower cumulative incidence rate [22]. Lorlatinib, an ALK third-generation TKI, displayed an intracranial response of 63% [23,24,25]. Lorlatinib was designed to be a CNS penetrant and was highly effective in treating CNS metastases throughout previous clinical studies [26]. Due to the increase in life expectancy for lung cancer patients resulting from advancements in chemotherapy, there have been numerous instances where multiple gamma knife surgeries (GKS) or surgeries were performed.

In our study, the use of targeted therapy, particularly from second- and third-generations, delivered a better PFS than other cytotoxic chemotherapies. Although there was no significant difference in PFS or OS, only 25 of 96 patients received brain local treatment after. The remaining patients either succumbed to lung cancer without receiving local brain treatment or are currently being followed up, with or without recurrence. It can be inferred that if asymptomatic small brain metastases were all treated upon initial diagnosis, unnecessary treatments would have been performed. Thus, asymptomatic brain metastases from lung cancer can be followed up without local treatments such as WBRT or SRS during the targeted therapy. However, a short-term follow-up for brain metastases is essential to evaluate the drug response. There was also no difference in the rates of brain-related deaths among the patients in our study (2%) and that of Venur VA. et al. (5%), which provides further evidence for this claim [27].

Nevertheless, our retrospective study has several limitations. Firstly, due to it being a study conducted at a single institution aimed at anticipating the effects of chemotherapy, it was not feasible to ascertain the results by referencing the period during which brain local treatment was administered to asymptomatic patients. Second, the study sample size might be small, potentially affecting the generalizability of the findings. If the number of participants is limited, it could compromise the statistical power and precision of the results. Moreover, the article does not provide information on the specific systemic therapies used or their dosages, which makes it difficult to assess the efficacy and compare the outcomes with other studies. Additionally, the study design is not specified, raising questions about the potential biases or confounding factors that might have influenced the results. Furthermore, the article lacks long-term follow-up data, making it challenging to evaluate the durability of the observed intracranial efficacy. Considering these limitations, further research with larger sample sizes, well-defined study designs, and longer follow-up periods are necessary to provide a comprehensive understanding of the intracranial efficacy of systemic therapy in patients with asymptomatic brain metastases from lung cancer.

## 5. Conclusions

Patients with brain metastases from lung cancer who receive treatment with second- and third-generation targeted therapies tend to experience longer PFS even in the absence of local brain treatments. In cases of asymptomatic brain metastases treated with targeted therapy, it is reasonable to opt for follow-up without local treatment. However, in situations where cytotoxic chemotherapy is administered without the presence of an EGFR or ALK mutation, more stringent follow-up procedures may be necessary to facilitate the implementation of additional treatments.

## Figures and Tables

**Table 1:** Clinical characteristics of 96 patients.

Clinical Variable		No. Patients (%)
Age (years)	≥70	55 (57.2%)
	<70	41 (43.8%)
Pathology	Adenocarcinoma	74 (77%)
	Squamous cell carcinoma	11 (11.5%)
	Small cell carcinoma	11 (11.5%)
Other organ metastases	Yes	90 (93.7%)
	No	6 (6.3%)
Location	Supratentorial	66 (68.7%)
	Infratentorial	8 (8.3%)
	Both	22 (23%)
Number of tumors	1	47 (48.9%)
	2 to 5	24 (25%)
	Over 5	25 (26%)
Systemic therapy	Targeted therapy	39 (40.6%)
	Cytotoxic chemotherapy	57 (59.3%)
TKI generation	1st generation	23 (23.9%)
	2nd and 3rd generation	16 (16.6%)
	No targeted therapy	57 (59.3%)
Recurrence pattern	No recurrence	44 (45.8%)
	Local	8 (8.3%)
	Distant and Local	41 (42.7%)
	Dissemination	3 (3.1%)

(TKI: tyrosine kinase inhibitor).

**Table 2:** Univariate analysis.

**PFS-Related Factors**			
**Clinical Factors**		**Median PFS** **(Min-Max)**	**PFS** ***p*-Value**
Age	≥70	7.7 (1.9–48.3)	0.861
	<70	6.2 (1.1–47.9)	
Pathology	Adenocarcinoma	7.9 (1.3–48.3)	0.069
	Squamous cell carcinoma	3.9 (1.1–20.5)	
	Small cell carcinoma	7.7 (2.2–25.7)	
Location	Supratentorial	7.4 (1.1–48.3)	0.614
	Infratentorial	8.2 (2.0–10.9)	
	Both	6.8 (1.9–47.9)	
Number	1	7.4 (1.3–48.3)	0.857
	2 to 5	6.6 (1.1–47.9)	
	Over 5	7.9 (1.9–36.2)	
Other organ metastases	Yes	7.2 (1.1–48.3)	0.399
	No	16.5 (3.4–47.9)	
Systemic therapy	Targeted therapy	10.2 (1.1–48.3)	0.020
	Cytotoxic chemotherapy	5.5 (1.7–47.9)	
TKI generation	1st generation	7.9 (1.1–42.8)	0.016
	2nd and 3rd generation	16.0 (2.0–48.3)	
	No targeted therapy	5.5 (1.7–47.9)	
**OS-related factors**			
**Clinical factors**		**median OS** **(min-max)**	**OS** ***p*-value**
Age	≥70	13.0 (2.0–65.0)	0.972
	<70	16.4 (3.4–60.2)	
Pathology	Adenocarcinoma	15.4 (2.0–65.0)	0.032
	Squamous cell carcinoma	6.7 (3.0–23.5)	
	Small cell carcinoma	13.1 (2.2–24.8)	
Location	Supratentorial	16.1 (2.2–65.0)	0.240
	Infratentorial	12.4 (5.1–25.9)	
	Both	12.4 (2.0–47.9)	
Number	1	18.2 (3.0–65.0)	0.124
	2 to 5	12.1 (2.0–47.9)	
	Over 5	11.8 (2.1–39.3)	
Other organ metastases	Yes	13.4 (2.0–60.2)	0.010
	No	20.2 (4.8–65.0)	
Systemic therapy	Targeted therapy	18.2 (3.0–51.3)	0.445
	Cytotoxic chemotherapy	13.1 (2.0–65.0)	
TKI generation	1st generation	14.0 (3.0–43.0)	0.401
	2nd and 3rd generation	18.2 (5.3–51.3)	
	No targeted therapy	13.1 (2.0–65.0)	
Recurrence pattern	No recurrence	15.7 (2.0–60.2)	0.208
	Local	15.8 (5.5–65.0)	
	Distant and Local	13.4 (3.4–51.3)	
	Dissemination	7.2 (3.9–10.4)	

(PFS: progression-free survival, OS: overall survival, TKI: tyrosine kinase inhibitor).

**Table 3:** Multivariate analysis.

**PFS-Related Factors**				
**Clinical Factors**		**Hazard Ratio**	***p*-Value**	**95% CI**
Other organ metastases	Absence Presence (reference)	0.347 1	0.068	0.111–1.080
Pathology	Adenocarcinoma Squamous cell carcinoma Small cell carcinoma (reference)	0.824 1.827 1	0.642 0.249	0.365–1.862 0.656–5.089
TKI generation	2nd and 3rd generation 1st generation No targeted therapy (reference)	0.229 0.740 1	0.005 0.395	0.082–0.640 0.370–1.480
**OS-related factors**				
**Clinical factors**		**Hazard ratio**	***p*-value**	**95% CI**
Other organ metastases	Absence Presence (reference)	0.094 1	0.020	0.013–0.689
Pathology	Adenocarcinoma Squamous cell carcinoma small cell carcinoma (reference)	0.608 1.479 1	0.205 0.412	0.281–1.313 0.580–3.772
TKI generation	2nd and 3rd generation 1st generation No targeted therapy (reference)	0.555 0.974 1	0.123 0.927	0.263–1.173 0.558–1.702
Recurrence pattern	No dissemination Dissemination	0.279 1	0.098	0.062–1.266

Reference variables: the presence of other organ metastases, small cell carcinoma, no targeted therapy, dissemination (PFS: progression-free survival, OS: overall survival, TKI: tyrosine kinase inhibitor).

## Data Availability

The data presented in this study are available on request from the corresponding author. The data are not publicly available due to due to privacy or ethical restrictions.
